# UDP-Galactose 4′-Epimerase Activities toward UDP-Gal and UDP-GalNAc Play Different Roles in the Development of *Drosophila melanogaster*

**DOI:** 10.1371/journal.pgen.1002721

**Published:** 2012-05-24

**Authors:** Jennifer M. I. Daenzer, Rebecca D. Sanders, Darwin Hang, Judith L. Fridovich-Keil

**Affiliations:** 1 Graduate Program in Genetics and Molecular Biology, Emory University, Atlanta, Georgia, United States of America; 2 Graduate Program in Biochemistry, Cell and Developmental Biology, Emory University, Atlanta, Georgia, United States of America; 3 Department of Human Genetics, Emory University School of Medicine, Atlanta, Georgia, United States of America; Harvard Medical School, United States of America

## Abstract

In both humans and *Drosophila melanogaster*, UDP-galactose 4′-epimerase (GALE) catalyzes two distinct reactions, interconverting UDP-galactose (UDP-gal) and UDP-glucose (UDP-glc) in the final step of the Leloir pathway of galactose metabolism, and also interconverting UDP-N-acetylgalactosamine (UDP-galNAc) and UDP-N-acetylglucosamine (UDP-glcNAc). All four of these UDP-sugars serve as vital substrates for glycosylation in metazoans. Partial loss of GALE in humans results in the spectrum disorder epimerase deficiency galactosemia; partial loss of GALE in *Drosophila melanogaster* also results in galactose-sensitivity, and complete loss in *Drosophila* is embryonic lethal. However, whether these outcomes in both humans and flies result from loss of one GALE activity, the other, or both has remained unknown. To address this question, we uncoupled the two activities in a *Drosophila* model, effectively replacing the endogenous *dGALE* with prokaryotic transgenes, one of which (*Escherichia coli GALE)* efficiently interconverts only UDP-gal/UDP-glc, and the other of which (*Plesiomonas shigelloides wbgU)* efficiently interconverts only UDP-galNAc/UDP-glcNAc. Our results demonstrate that both UDP-gal and UDP-galNAc activities of *dGALE* are required for *Drosophila* survival, although distinct roles for each activity can be seen in specific windows of developmental time or in response to a galactose challenge. By extension, these data also suggest that both activities might play distinct and essential roles in humans.

## Author Summary

In this manuscript we apply a fruit fly model to explore the relative contributions of each of two different activities attributed to a single enzyme—UDP-galactose 4′-epimerase (GALE); partial impairment of human GALE results in the potentially severe metabolic disorder epimerase deficiency galactosemia. One GALE activity involves interconverting UDP-galactose and UDP-glucose in the Leloir pathway of galactose metabolism; the other activity involves interconverting UDP-N-acetylgalactosamine and UDP-N-acetylglucosamine. We have previously demonstrated that complete loss of GALE is embryonic lethal in fruit flies, but it was unclear which GALE activity loss was responsible for the outcome. Using genetically modified fruit flies, we were able to remove or give back each GALE activity individually at different times in development and observe the consequences. Our results demonstrate that both GALE activities are essential, although they play different roles at different times in development. These results provide insight into the normal functions of GALE and also have implications for diagnosis and intervention in epimerase deficiency galactosemia.

## Introduction

Galactose is an essential component of glycoproteins and glycolipids in metazoans, and as a constituent monosaccharide of the milk sugar, lactose, also serves as a key nutrient for mammalian infants. Galactose is also found in notable quantities in some fruits, vegetables, and legumes. Galactose is both synthesized and catabolized in all species via the Leloir pathway, which is highly conserved across branches of the evolutionary tree [1].

**Figure 1:**
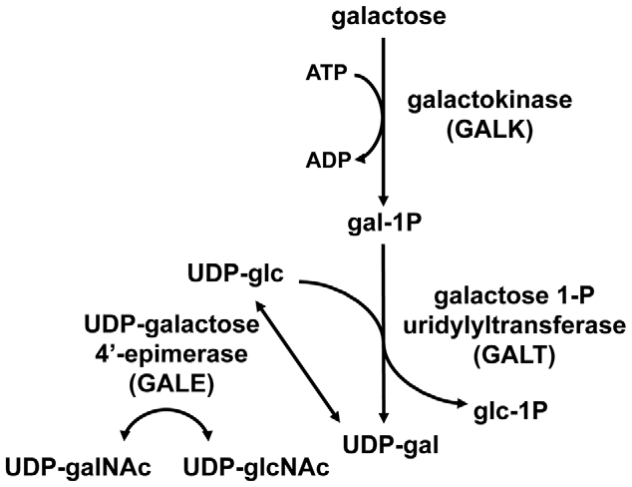
The Leloir pathway of galactose metabolism. UDP-galactose 4′-epimerase, the third enzyme in the pathway, also interconverts UDP-N-acetylgalactosamine (UDP-galNAc) and UDP-N-acetylglucosamine (UDP-glcNAc) in humans, *Drosophila*, and other metazoans tested.

The reactions of the Leloir pathway are catalyzed by the sequential activities of three enzymes: (1) galactokinase (GALK) which phosphorylates alpha-D-galactose to form galactose-1-phosphate (gal-1P), (2) galactose-1-phosphate uridylyltransferase (GALT), which transfers uridine monophosphate (UMP) from uridine diphosphoglucose (UDP-glc) to gal-1P, forming UDP-galactose (UDP-gal) and releasing glucose-1-phosphate (glc-1P), which can proceed to phosphoglucomutase and the glycolytic pathway, and (3) UDP-galactose 4′-epimerase (GALE) which interconverts UDP-gal and UDP-glc [1]. In addition to a role in the Leloir pathway, metazoan GALE enzymes also interconvert UDP-N-acetylgalactosamine (UDP-galNAc) and UDP-N-acetylglucosamine (UDP-glcNAc) (Figure 1). Because it catalyzes reversible reactions, GALE therefore not only contributes to the catabolism of dietary galactose, but also enables the endogenous biosynthesis of both UDP-gal and UDP-galNAc [2], [3] when exogenous sources are limited.

Deficiency in any of the three Leloir enzymes in humans results in a form of the metabolic disorder galactosemia, although the symptoms and clinical severity differ according to which enzyme is impaired and the extent of the impairment. Profound loss of hGALE results in generalized epimerase-deficiency galactosemia, an autosomal recessive and potentially severe disorder. To date, however, no patient has been reported with complete loss of GALE, and even the most severely affected demonstrate at least 5% residual enzyme activity [4]. Previous studies have indicated that different patient mutations impair hGALE to different extents [5]–[9]. Further, while some mutations impair both GALE activities similarly, others do not. For example, the *hGALE* allele V94M, which leads to severe epimerase-deficiency galactosemia in the homozygous state, encodes an enzyme that retains ∼5% residual activity toward UDP-gal but ∼25% residual activity toward UDP-galNAc [8], [9]. Disparities such as this have raised the question of whether the pathophysiology of epimerase deficiency galactosemia results from the loss of GALE activity toward UDP-gal/UDP-glc, or toward UDP-galNAc/UDP-glcNAc, or both.

To address this question, we applied a *Drosophila melanogaster* model of *GALE* deficiency [10]. Using this model, we have previously established that *GALE* is essential in *Drosophila*; animals completely lacking endogenous *dGALE* succumb as embryos, and conditional loss of *dGALE* in larvae results in death within two to four days of knockdown. Finally, partial loss of *dGALE* leads to galactose sensitivity in larvae, and transgenic expression of human *GALE* (*hGALE*) rescues each of these negative outcomes [7].

Here we have applied our transgenic *Drosophila* model to uncouple and examine the individual roles of GALE separately. Toward that end, we generated flies that lacked endogenous *dGALE* and expressed either of two prokaryotic transgenes, one encoding *E. coli* GALE (*eGALE)* which exhibits an approximately 8,000-fold substrate preference for UDP-gal/UDP-glc over UDP-galNAc/UDP-glcNAc [11], and the other encoding *P. shigelloides wbgU*, which exhibits an approximately 2,000-fold substrate preference for UDP-galNAc/UDP-glcNAc over UDP-gal/UDP-glc [12]. By expressing these prokaryotic transgenes individually or in combination in *dGALE*-deficient *Drosophila* we determined that both GALE activities are required for survival of embryos and larvae. We also found that restoration of one activity or the other in later development rescued some phenotypes. Combined, these results provide insight into the varied roles of *dGALE* in *Drosophila* development and homeostasis, and by extension, suggest that *hGALE* may play similarly complex and essential roles in humans.

## Results

### *The Drosophila* GALE enzyme efficiently interconverts both UDP-gal/UDP-glc and UDP-galNAc/UDP-glcNAc

**Figure 2:**
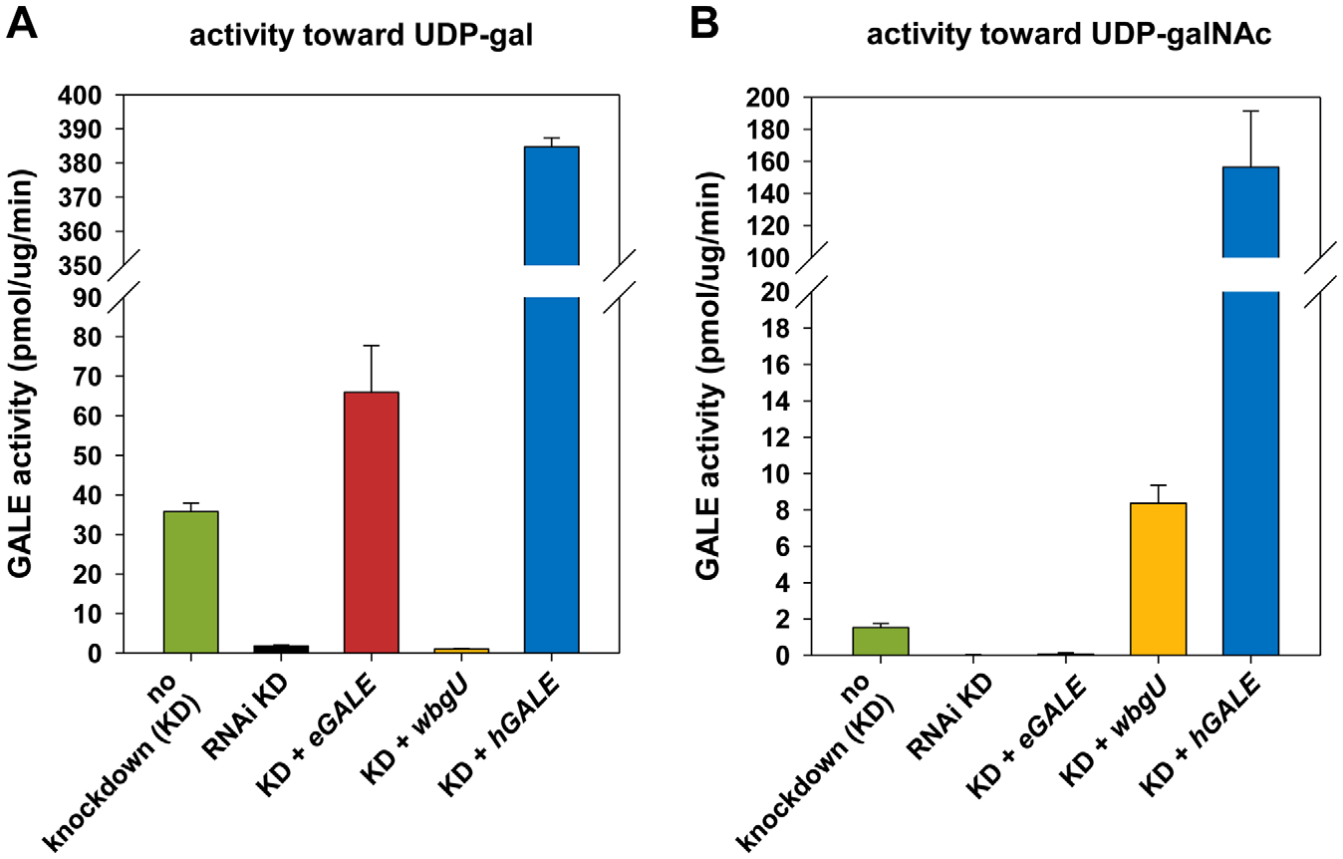
Enzyme activities of flies expressing different *GALE* **transgenes.** Assays for all genotypes were performed on flies with *dGALE* knockdown (KD) driven by the *Act5C-GAL4* driver with the exception of flies labeled “no knockdown”; those flies carried the same *UAS-RNAi^dGALE^* and *GAL80^ts^* alleles, but were balanced over TSTL, and thus lacked the driver. In addition to RNA^i^ knockdown of *dGALE*, *Act5C-GAL4* also drives expression of the specified transgenes in these animals. Panel A: GALE activity using UDP-gal as substrate. Panel B: GALE activity using UDP-galNAc as substrate.

Human and other mammalian GALE enzymes efficiently interconvert both UDP-gal/UDP-glc and UDP-galNAc/UDP-glcNAc (e.g. [13]–[15]). Previously, we reported that *Drosophila* GALE interconverts the first of these substrate pairs (UDP-gal/UDP-glc) [10], but did not address whether dGALE could also interconvert the second. Here we demonstrate that dGALE from wild-type adult flies efficiently interconverts both substrate sets (left most bar, Figure 2). Of note, while purified human GALE [15] and dGALE each interconvert both UDP-gal/UDP-glc and UDP-galNAc/UDP-glcNAc, the apparent specific activity of both human and fly enzymes toward UDP-gal is significantly higher than toward UDP-galNAc.

### *eGALE* and *wbgU* transgenes enable the expression of individual GALE activities in *Drosophila*

To generate flies with epimerase activity toward only UDP-gal/UDP-glc or only UDP-galNAc/UDP-glcNAc, we created transgenic lines expressing *eGALE* (*UAS-eGALE*) or *wbgU* (*UAS-wbgU*), respectively, each in a conditionally *dGALE*-impaired background. Each of these prokaryotic *GALE* genes has been demonstrated previously to encode epimerase activity toward only one of the two sets of epimer pairs (e.g. [11], [12]). To minimize background, activities of the encoded eGALE and WbgU enzymes toward UDP-gal and UDP-galNAc were assayed in flies knocked down for endogenous *dGALE*; results for the transgenes that demonstrated activities closest to those seen in wild-type *Drosophila, eGALE^62A^ and wbgU^19A^,* are presented in Figure 2. As expected, lysates from *dGALE* knockdown flies expressing the *eGALE* transgene demonstrated strong activity toward UDP-gal, but not UDP-galNAc, and lysates from *dGALE* knockdown flies expressing the *wbgU* transgene demonstrated strong activity toward UDP-galNAc, but not UDP-gal. As a control we also tested lysates from *dGALE* knockdown flies expressing a human *GALE* transgene; as expected, those samples demonstrated very strong activity toward both substrates.

### Both *eGALE* and *wbgU* transgene activities are required, in combination, to rescue viability of *dGALE*–deficient *Drosophila*

Previously, we created and characterized two *dGALE*-deficient alleles, *dGALE^f00624.4^* and *dGALE^Δy^*, which allowed us to demonstrate that *GALE* is essential for survival in *Drosophila* [10]. To examine the requirement for the two different epimerase activities separately, we set up crosses which allowed for the expression of *eGALE* or *wbgU*, individually or in combination, driven by *Act5C-GAL4* in an otherwise *dGALE*-deficient background (*dGALE^f00624.4^/dGALE^Δy^*). Table 1 shows the observed to expected ratios of surviving transgenic offspring that eclosed from these crosses. As presented in Table 1, neither *eGALE* alone nor *wbgU* alone was sufficient to rescue survival of the *dGALE*-deficient animals; however, expression of both *eGALE* and *wbgU*, in combination, was sufficient. These results demonstrate that GALE activities toward both UDP-gal and UDP-galNAc are essential for survival of *D. melanogaster*. To rule out the possibility that rescue with *eGALE* plus *wbgU* in combination occurred not because both GALE activities are essential but rather because neither individual transgene expressed sufficient enzyme, we also tested additional *eGALE* and *wbgU* transgenes that individually demonstrated higher levels of expression; none was sufficient to rescue (data not shown). Of note, there also was no apparent over-expression phenotype; for example, animals expressing either *eGALE* or *wbgU* in *addition* to endogenous *dGALE*, and animals dramatically over-expressing human GALE, remained viable, fertile, and appeared morphologically normal (data not shown).

**Table 1:** Crosses to test rescue of *wbgU* and *eGALE* transgenes individually and in combination.

experimental cross	genotype	Expected mendelian proportion of F1 with this genotype	Observed proportion of viable F1 with this genotype
*Actin5C-GAL4*/*CyO* ; *dGALE^y^*/*TM6B*×*Actin5C-GAL4*/*CyO* ; *UAS-wbgU*, *dGALE^f00624.4^*/*TM6B*	*Actin5C-GAL4/CyO ; UAS-wbgU*, *dGALE^f00624.4^*/*dGALE^y^*	0.333	0.000±0.000
*Actin5C-GAL4/CyO ; dGALE^y^/TM6B*×*UAS-eGALE/CyO ; dGALE^f00624.4^/TM6B*	*Actin5C-GAL4/UAS-eGALE; dGALE^f00624.4^*/*dGALE^y^*	0.143	0.000±0.000
*UAS-eGALE/CyO ; dGALE^y^*/*/TM6B*×*Actin5C-GAL4/CyO ; UAS-wbgU*,*dGALE^f00624.4^/TM6B*	*Actin5C-GAL4/UAS-eGALE; UAS-wbgU*,*dGALE^f00624.4^*/*dGALE^y^*	0.143	0.176±0.018

### Different requirements for GALE activities at different stages of *Drosophila* development

**Figure 3:**
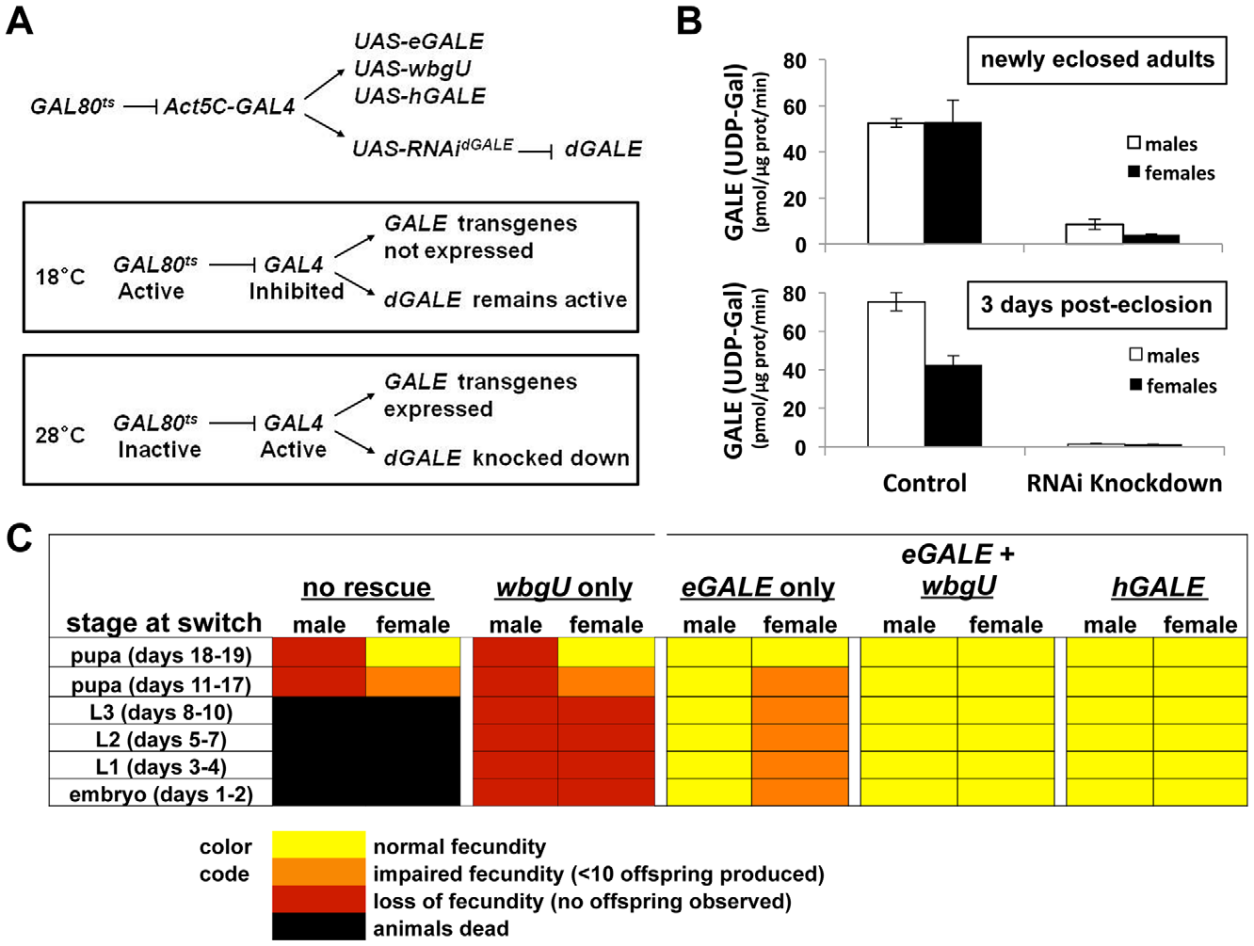
Differentially impaired fecundity of flies lacking different GALE activities. (A) Diagram of the method used to achieve expression of different *GALE* transgenes in the background of *dGALE* knockdown animals. The timing of knockdown and concurrent transgene expression was controlled by switching flies from the permissive temperature (18°C) to the restrictive temperature (28–29°C), as indicated. (B) Knockdown efficiency in male and female animals switched to the restrictive temperature as early to mid-stage pupa and harvested for biochemical analysis as newly eclosed adults or three days after eclosion. Of note, GALT activity was completely normal in all samples tested and apparently unaffected by the *dGALE* knockdown (data not shown). (C) Each box represents the outcome of flies switched from 18°C to 28°C at the stage indicated in the column on the left. The number of days the flies developed at 18°C to reach each stage is shown in parentheses.

Previously, we described an approach that achieves conditional knockdown of *dGALE* in *Drosophila* using a *UAS-RNAi^dGALE^* transgene (12030-R2, National Institute of Genetics Fly Stock Center, Mishima, Shizuoka, Japan) in combination with a temperature sensitive allele of yeast *GAL80* (*GAL80^ts^*) ([10] and Figure 3A). Using this system, we found that *dGALE* is required from embryogenesis through pupation, and that loss of *dGALE* during pupation leads to defects in fecundity and perhaps also a shortened life span [10].

Here we have expanded the *GAL80^ts^*conditional *dGALE* knockdown system to include different *GAL4*-dependent *GALE* transgenes and have applied this expanded system to test the ability of each transgene, or pair of transgenes, to compensate for the loss of endogenous *dGALE*. By using age-synchronized cohorts of animals and shifting from the permissive (18°C) to the restrictive temperature (28–29°C) at different times we also were able to test the ability of each *GALE* transgene, or pair of transgenes, to sustain survival and fecundity at different stages of development. At 18°C these animals expressed endogenous *dGALE*, but not their transgenes, and at 28–29°C these animals expressed their transgenes but not *dGALE* (Figure 3A). Specifically, we tested *Drosophila* that carried no *GALE* transgene, an *eGALE* transgene, a *wbgU* transgene, both *eGALE* and *wbgU* transgenes, or an *hGALE* transgene.

As expected from prior results ([10] and Table 1), animals expressing no *GALE* transgene succumbed when shifted to the restrictive temperature as larvae, while animals expressing either human *GALE* or both *eGALE* plus *wbgU* remained viable and fertile (Figure 3C). Surprisingly, expression of either *eGALE* or *wbgU* alone was also sufficient to rescue survival, albeit to a lesser extent. The fact that the individual prokaryotic transgenes were sufficient to rescue *dGALE* knockdown animals, but not animals genetically null for *dGALE* (Table 1), suggests that trace residual *dGALE* expression in the knockdown animals lowered the threshold of transgene function required for rescue.

Of note, while *dGALE* knockdown animals encoding either *eGALE* or *wbgU* remained viable following a shift to the restrictive temperature in early to mid-development (Figure 3C), these survivors were not entirely healthy. Specifically, these animals demonstrated either partial or complete loss of fecundity as adults. To test whether the degree of *dGALE* knockdown was comparable between males and females, and therefore not a confounding factor in differential outcome, we performed GALE and GALT enzyme assays on newly eclosed and three day old male and female knockdown adults that carried no GALE transgene and that had been switched to the restrictive temperature as early to mid-stage pupa. The degree of GALE knockdown in both males and females was profound and comparable (Figure 3B). As expected, the level of GALE activity was even lower in the older animals, presumably because any GALE synthesized prior to the temperature switch had three additional days to decay. Also as expected, GALT activity was normal and apparently unaffected by the *dGALE* knockdown in all samples tested (data not shown).

To examine fecundity, we collected and sequestered newly eclosed virgin female and male flies from each surviving cohort, crossed them to an equal number of wild-type flies of the opposite sex, and counted the numbers of viable offspring resulting from each cross. Crosses resulting in large numbers of viable offspring (>50) were scored as “normal fecundity”. Crosses resulting in fewer than 10 viable offspring were scored as “reduced fecundity,” and crosses resulting in no viable offspring were scored as “loss of fecundity” (Figure 3C). For example, when *dGALE* knockdown was initiated during early to mid-stage pupal development, animals of both sexes displayed diminished fecundity. Expression of *eGALE* alone, but not *wbgU* alone, rescued the male defect, whereas expression of both prokaryotic transgenes in combination, or *hGALE* alone, was required to rescue the female defect. These results indicate that GALE activity toward UDP-gal is both necessary and sufficient for male fecundity, but that GALE activities toward both UDP-gal and UDP-galNAc are required for female fecundity.

### Galactose exposure of transgenic flies with late-onset *dGALE* knockdown reveals differential roles of GALE activities toward UDP-gal and UDP-galNAc

We have previously demonstrated that *Drosophila* expressing a hypomorphic allele of *dGALE* are viable but sensitive to galactose exposure [10]. To assess the roles of the two *GALE* activities in coping with environmental galactose, we collected adult flies in which *dGALE* knockdown coupled with *hGALE*, *eGALE*, *wbgU*, or *eGALE* plus *wbgU* transgene expression was initiated using the *GAL80^ts^* system during late larval or early-to-mid-pupal development. These animals were allowed to develop on a standard molasses-based food, and were then transferred as newly eclosed adults to food containing either 555 mM glucose as the sole sugar, or 555 mM glucose plus 175 mM galactose.

**Figure 4:**
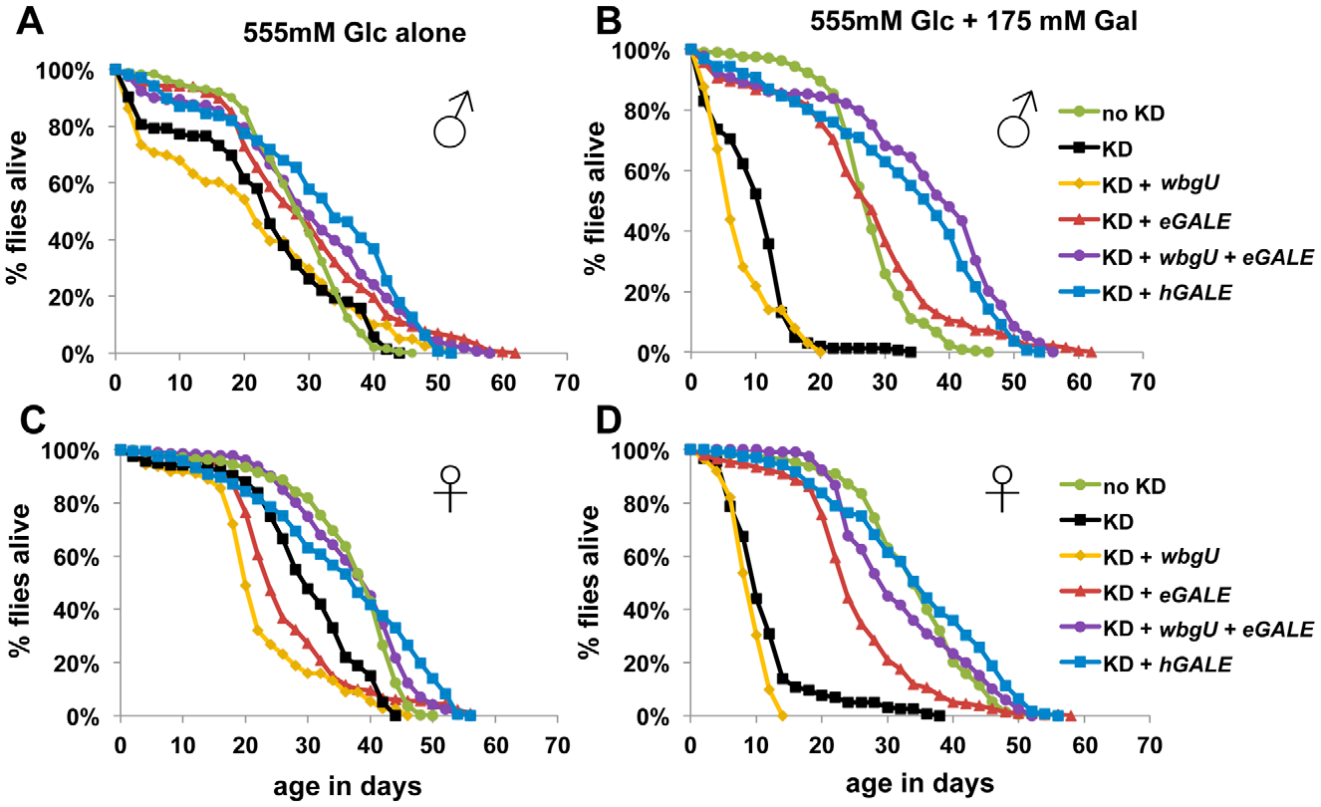
Flies lacking GALE activity toward UDP-gal/UDP-glc have a shortened life span when exposed to galactose as adults. The life spans of male (A and B) and female (C and D) flies reared on molasses food and then tapped as newly eclosed adults to food containing either 555 mM glucose only (A and C), or 555 mM glucose plus 175 mM galactose (B and D), is illustrated. As indicated by the key, these cohorts of flies included controls expressing endogenous *dGALE* as well as animals that expressed endogenous *dGALE* early in development but then were subjected late in development to *dGALE* knockdown coupled with induced expression of either no *GALE* transgene, or *wbgU*, *eGALE*, *hGALE*, or both *wbgU* and *eGALE* in combination. Based on Log rank and Wilcoxon tests for significance, the life spans of knockdown animals expressing either no transgene or expressing only *wbgU* were significantly decreased on food containing galactose compared with food containing only glucose (p<0.0001).

We assessed the lifespan of each cohort of animals on both foods; as a control, knockdown animals expressing no *GALE* transgene were also monitored (Figure 4). In the absence of galactose, all cohorts showed similar longevity profiles, although females (Figure 4C) showed greater variability than males (Figure 4A). In the presence of galactose, however, both males and females expressing either no *GALE* transgene, or only the *wbgU* transgene, demonstrated a dramatic reduction in life span (p<0.0001, Figure 4B and 4D). Females expressing *eGALE* alone exhibited a slight decrease in life span that was independent of diet. Animals expressing *hGALE* or *eGALE*+*wbgU* had lifespans comparable to control animals expressing endogenous *dGALE*, regardless of diet. These data implicate loss of UDP-gal activity as responsible for the galactose-dependent early demise of adult *dGALE*-impaired *Drosophila*.

### Differentially *GALE*–impaired flies exposed to galactose demonstrate different metabolic abnormalities

**Figure 5:**
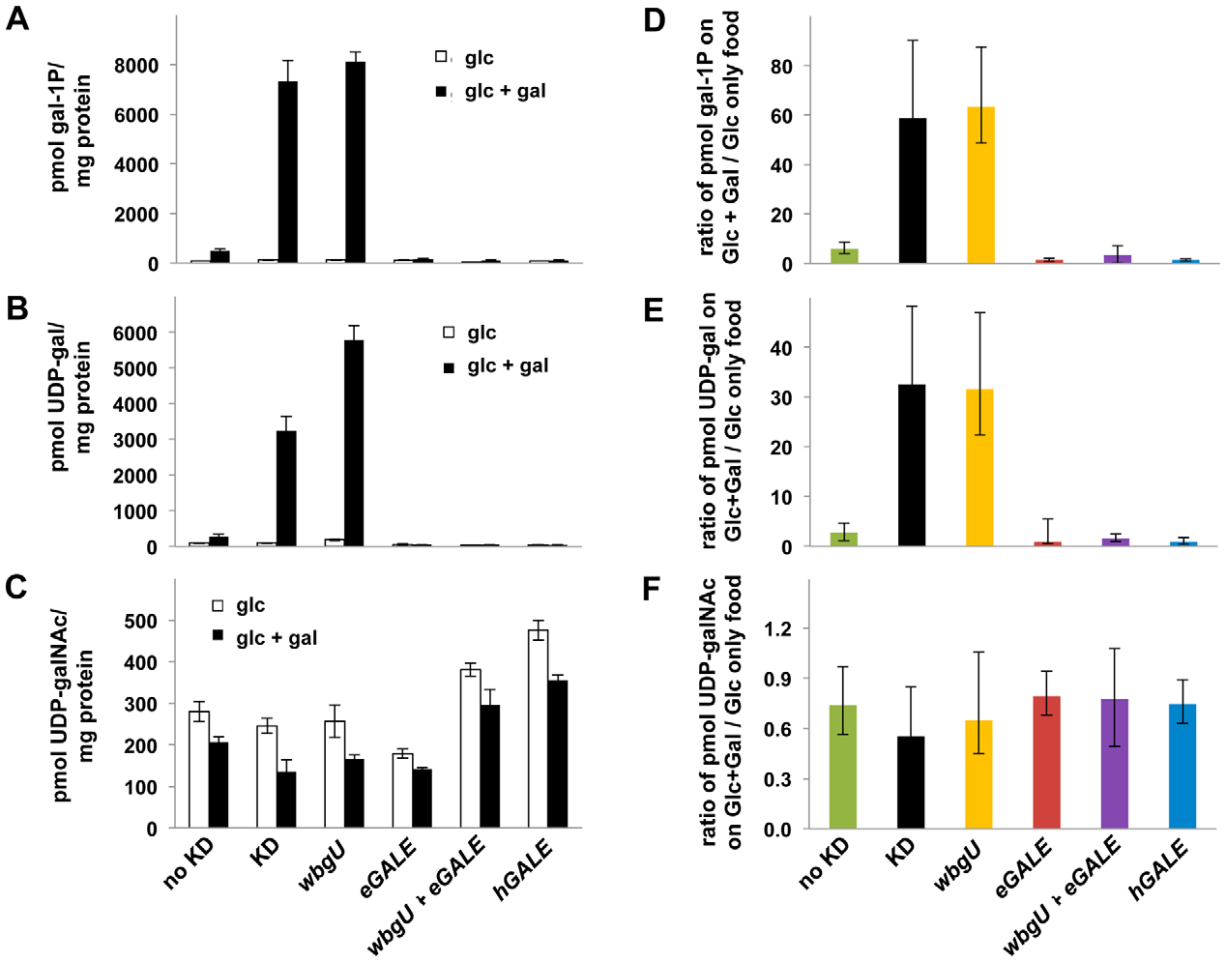
Metabolite profiles of *Drosophila* exposed to galactose. Metabolites were extracted from cohorts of larvae raised on food containing either 555 mM glucose or 555 mM glucose+175 mM galactose. Animals were shifted from the permissive temperature (18°C) to the restrictive temperature (28°C) as first instar larvae and allowed to develop for four days before harvest. Accumulated metabolite values for gal-1P (A), UDP-gal (B), and UDP-galNAc (C) are shown on food containing glucose and glucose+galactose. To demonstrate the impact of diet on metabolite levels, values for gal-1P (D), UDP-gal (E), and UDP-galNAc (F) are shown as ratios of the amount of each metabolite accumulated by animals on food containing galactose over that accumulated by animals of the same genotype on food containing only glucose. Error bars show the 95% confidence interval for each ratio.

As one approach to explore the pathophysiology underlying the different galactose-dependent outcomes observed in *Drosophila* deficient in GALE activity toward UDP-gal or UDP-galNAc we measured the levels of gal-1P, UDP-gal, and UDP-galNAc in lysates prepared from galactose-exposed third instar larvae expressing different *GALE* transgenes. As illustrated in Figure 5, galactose exposed animals deficient in both GALE activities (bars marked “KD” for knockdown) accumulated abnormally high levels of gal-1P (Figure 5A and 5D) and UDP-gal (Figure 5B and 5E). Animals deficient only in GALE activity toward UDP-gal (bars marked “*wbgU*” in Figure 5) also demonstrated elevated gal-1P (Figure 5A and 5D) and UDP-gal (Figure 5B and 5E). In contrast, galactose exposed larvae deficient only in GALE activity toward UDP-galNAc (bars marked “*eGALE*” in Figure 5C and 5F) demonstrated no extraordinary metabolic abnormalities, although, as expected, the absolute level of UDP-galNAc was diminished in these animals independent of diet relative to the “no knockdown” control (Figure 5C). Also as expected, animals expressing either *hGALE* or both *eGALE* plus *wbgU* demonstrated no clear metabolic abnormalities (Figure 5).

## Discussion

UDP-galactose 4′-epimerase (GALE) is an essential enzyme in *Drosophila* [10] and in humans [16], but until now the relative contributions of the two distinct GALE activities to development and galactose-tolerance has remained unclear. Understanding these roles has important implications regarding mechanism of galactose sensitivity, and may be applicable to diagnosis and prognosis in humans with epimerase-deficiency galactosemia. Our experiments described here exploit the genetic and biochemical facility of *Drosophila melanogaster* to test the consequences of losing each of the two GALE activities individually at different stages of development, or under different conditions of galactose exposure.

Our results demonstrate that developing animals require at least some GALE activity toward both epimer pairs, even in the absence of dietary galactose. Complete loss of either activity in embryos is lethal (Table 1). In animals with trace dGALE activity left by knockdown rather than genetic deletion or disruption, however, transgenic expression of either GALE activity alone is sufficient for rescue (Figure 3C). Further, in animals that expressed both GALE activities as larvae, knockdown of both activities during pupation is not lethal.

However, knockdown of either GALE activity in early development, or knockdown of both activities in later development has consequences. For example, loss of activity toward UDP-gal in larvae results in impaired fecundity of both males and females, while loss of activity toward UDP-galNAc in larvae results in impaired fecundity of females but not males. Individual loss of one activity or the other later in development also results in differential sensitivity to galactose. Specifically, both male and female flies deficient in GALE activity toward UDP-gal exhibit a markedly reduced lifespan when exposed to galactose; this effect is not seen in wild-type flies or in flies uniquely deficient in GALE activity toward UDP-galNAc.

These results support two important conclusions. First, the essential role of GALE in development and homeostasis of *Drosophila* extends beyond the Leloir pathway. Whether GALE activity toward UDP-galNAc is essential because of its presumed role in establishing and maintaining substrate pools for glycosylation, or for some other reason, remains unknown. Prior studies in GALE-deficient mammalian cells [17] showed that uridine supplementation could rescue growth and some metabolic abnormalities caused by galactose exposure, raising the possibility that depleted pools of uridine or uridine-derivatives might also be contributing factors. In the current study it is also unclear whether animals subjected to knockdown of one or both GALE activities later in development demonstrate a less severe outcome than those knocked down earlier in development because the products of GALE function, namely UDP-gal, UDP-glc, UDP-galNAc, and UDP-glcNAc, are less essential later in development, or rather because these UDP sugars have already accumulated to sufficient levels and can be recycled for use. Similarly, the differential sensitivities of male and female fecundity to loss of GALE activity later in development may reflect fundamental differences in male and female development, or alternatively may reflect differential sensitivity to loss; for example, eggs may require a more substantial pool of specific UDP-sugar substrates than sperm to give rise to a viable embryo.

### Implications for mechanism

The disparate metabolic profiles observed in GALE-impaired flies exposed to galactose provide a window of insight into potential mechanisms behind the outcomes observed. For example, gal-1P accumulates to abnormal levels in animals missing GALE activity toward UDP-gal but not UDP-galNAc, and only those animals demonstrate substantially reduced lifespan when exposed to galactose as adults. This metabolic result is expected, since only GALE activity toward UDP-gal should impact the Leloir pathway, and this outcome result implies that gal-1P might contribute to the early demise of these animals. However, the gal-1P result also implies that the negative outcomes observed in *Drosophila* deficient in GALE activity toward UDP-galNAc, e.g. compromised survival in embryos and compromised fecundity in adult females, do not result from gal-1P accumulation. This is an important point because it challenges the common supposition that gal-1P underlies pathophysiology in both classic and epimerase deficiency galactosemias. Clearly there must be another basis for the negative outcomes observed in these animals. It is also interesting to note that while loss of GALE activity toward UDP-galNAc in developing animals has phenotypic consequences, at least for female fecundity, it does not appear to negatively impact the “global” level of UDP-galNAc in animals exposed to galactose. The explanation for this apparent disparity might involve subtle or tissue-specific differences below the threshold of detection of our experimental approach.

### Implications for patients

The implications of this work for patients with epimerase deficiency galactosemia are two-fold. First, these results demonstrate that both GALE activities are essential for health of flies, and possibly also people. To our knowledge clinical laboratories that test patient samples for GALE activity only test activity toward UDP-gal. While this practice is certainly understandable, given that mutations may impact the two GALE activities differently [18]–[20], the results presented here raise the possibility that rare patients with GALE deficiency limited to UDP-galNAc activity could be missed. Second, given the impact of GALE-loss on both male and female fecundity in flies, these results suggest that long-term studies of both male and female reproductive issues in epimerase-deficiency galactosemia patients might be warranted.

## Materials and Methods

### Drosophila stocks and maintenance

The Drosophila stocks used in this study are listed in Table S1. Stocks were maintained at 25°C on a molasses-based food that contained 43.5 g/l cornmeal, 17.5 g/l yeast extract, 8.75 g/l agar, 54.7 ml/l molasses, 10 mls propionic acid and 14.4 ml/l tegosept mold inhibitor (10% w/v in ethanol). For experiments in which the levels and types of sugar were to be varied, we used a glucose-based food [5.5 g/l agar, 40 g/l yeast, 90 g/l cornmeal, 100 g/l glucose, 10 ml/l propionic acid and 14.4 ml/l tegosept mold inhibitor (10% w/v in ethanol)] [21] supplemented with galactose, as indicated.

### Generation of transgenic lines

*UAS*-*eGALE* and *UAS-wbgU* transgenes were generated by subcloning the *eGALE* and *wbgU* coding sequences, respectively, as *Eco*RI/*Xho*I fragments, into *pUAST* [22] using the *Eco*RI and *Xho*I sites in the *pUAST* polylinker region. The *wbgU* sequence was amplified from a plasmid generously provided by Peng George Wang (Ohio State University). Resulting plasmids were confirmed by sequence analysis. *UAS*-*eGALE* stocks were generated using standard transgenic techniques following injection of the transgene into embryos by the fly core of the Massachusetts General Hospital, Charlestown, MA. *UAS-wbgU* stocks were generated using standard transgenic techniques following injection of the transgene into embryos by Genetic Services, Inc., Cambridge, MA. Transformants were selected by the presence of the white gene within *pUAST*. Expression of functional *eGALE* or *wbgU* was confirmed by enzymatic assay of lysates from transformants.

### *GALK*, *GALT*, and *GALE* (UDP-gal) enzyme assays

Lysates were prepared and assays for GALK, GALT and GALE with UDP-gal as the substrate were performed (n≥3) as described previously [10].

### GALE assay conditions for activity toward UDP-galNAc

Activity was calculated from the conversion of UDP-galNAc to UDP-glcNAc. The initial reaction mixture concentrations were: 100 mM glycine pH 8.7, 1.6 mM UDP-galNAc and 0.5 mM NAD. Enzyme assays were performed as described in Sanders et al. [10] except for the following changes: To start each reaction, 7.5 µl of diluted protein and 5 µl of a cocktail of substrates and cofactors were combined. Reaction mixtures were incubated at 25°C for 30 minutes and then quenched by the addition of 112.5 µl of ice-cold high-performance liquid chromatography (HPLC)-grade water (Fisher). Lysates were diluted 1∶4, except for those prepared from animals with RNAi knockdown, which were undiluted, and those prepared from animals overexpressing *hGALE* or *wbgU* transgenes, which were diluted to a greater extent. Lysates from *Act5C>hGALE^22C^* animals were diluted 1∶60. Lysates from *Act5C>wbgU^19A^* animals were diluted 1∶20.

### Determining requirement for *GALE* activities in development and homeostasis

Generation of animals in which *GALE* knockdown was initiated at 24-hour intervals throughout development was achieved as described previously [10]. A stock homozygous for both *P{tubP-GAL80^ts^}10* and *12030R-2* was used in all crosses. These flies were then crossed to the appropriate genotypes to obtain offspring expressing various transgenes; for: no transgene, *P{Act5C-GAL4}25FO1 ; +/T(2;3)TSTL, Tb, Hu; eGALE only*, *P{Act5C-GAL4}25FO1, UAS-eGALE^62A^/CyO*; *wbgU* only, *P{Act5C-GAL4}25FO1/CyO; P{Act5C-GAL4}25FO1/CyO ; UAS-wbgU^19A^*/TM6B; *eGALE* plus *wbgU*, *P{Act5C-GAL4}25FO1, UAS-eGALE^62A^/CyO* ; *UAS-wbgU^19A^*/TM6B; *hGALE*, *P{Act5C-GAL4}25FO1/CyO ; UAS-hGALE^22C^/TM6B*. Adult flies eclosing from the vials were scored for the presence or absence of humeral and/or curly, as appropriate for each cross.

### Measurement of life span

Animals in which *dGALE* knockdown with concurrent transgene expression was achieved throughout development were obtained as described above. These animals were maintained on standard molasses medium until eclosion. Within 24 hours after eclosion, approximately 20 virgin male or female flies were placed in fresh vials of food containing 555 mM glucose only or 555 mM glucose plus 175 mM galactose. Flies were transferred to fresh food every 2–3 days, and the number of dead flies in each vial was recorded every other day. Log rank and Wilcoxon tests were used for statistical analysis using the program JMP (http://www.jmp.com/).

### Measuring metabolite accumulation in GALE–deficient larvae

Cohorts of newly hatched larvae raised at 18°C were transferred to vials of food containing either 555 mM glucose only or 555 mM glucose plus 175 mM galactose. The larvae were maintained at 18°C for one additional day, then transferred to 28°–29°C and allowed to develop for another four days prior to harvest. Metabolites were extracted and quantified as described previously [10], and were separated and quantified using a Dionex HPLC, as described previously [23] with the following changes: UDP-gal and UDP-galNAc were separated using a high salt isocratic procedure with a flow rate of 0.5 mL/min and buffer concentrations of 45% A and 55% B (0–61 min), followed by washing with a linear increase of B to 95% (61–80 min). For all samples, 20 µl were injected into a 25 µl injection loop. Ratios of the level of each metabolite on food containing galactose over the level on food containing glucose only were calculated, and 95% confidence intervals were determined using Fieller's theorem.

## Supporting Information

Table S1 *D. melanogaster* stocks and alleles used in this study.
(DOCX)
