# Supplementary material for: UDP-Galactose 4′-Epimerase Activities toward UDP-Gal and UDP-GalNAc Play Different Roles in the Development of Drosophila melanogaster
Source: PLoS Genet. 2012 May 24;8(5):e1002721. doi: 10.1371/journal.pgen.1002721 (PMC3359975; doi:10.1371/journal.pgen.1002721)
Supplement: Table S1 — D. melanogaster stocks and alleles used in this study. (DOCX) [file pgen.1002721.s001.docx]

**Table S1: *D. melanogaster* stocks and alleles used in this study.**

| **Fly stock or allele name** | **Comments** |
| --- | --- |
| *w^1118^* | Wild-type *D. melanogaster* (FBst0005905) |
| *dGALE^y^* | Imprecise excision of *P{EPgy2}CG12030^EY22205^* (FBst0022544) |
| *dGALE^f00624.4^* | P-element insertion *PBac{WH}CG12030^f00624^*in second intron of *dGALE* (FBst1016354), Harvard Exelixis Collection |
| *12030R-2* | UAS-RNAi to *dGALE* (National Institute of Genetics Fly Stock Center, Mishima, Shizuoka, Japan NM_138200.2) |
| *w*; P{tubP-GAL80ts}20;TM2/TM6B, Tb1* | Temperature sensitive allele of *GAL80* (FBst0007019) |
| *y1 w*; P{Act5C-GAL4}25FO1/CyO, y+* | *Actin5C-GAL4* driver used for rescue of *dGALE* loss and for *dGALE* knockdown (FBst0004414) |
| *eGALE^62A^* | *UAS-eGALE* insertion allele, chr II, homozygous lethal |
| *wbgU^19A^* | *UAS-wbgU* insertion allele, chr III, homozygous lethal |
| *hGALE^22C^* | *UAS-hGALE* insertion allele, chr III, homozygous viable |
